# Automated Retinal Layer Segmentation in *CLN2*-Associated Disease: Commercially Available Software Characterizing a Progressive Maculopathy

**DOI:** 10.1167/tvst.10.8.23

**Published:** 2021-07-27

**Authors:** Kyle D. Kovacs, Anton Orlin, Dolan Sondhi, Stephen M. Kaminsky, Donald J. D'Amico, Ronald G. Crystal, Szilárd Kiss

**Affiliations:** 1 Department of Ophthalmology, Retina Service, Weill Cornell Medical College, New York, NY, USA; 2 Department of Genetic Medicine, Weill Cornell Medical College, New York, NY, USA

**Keywords:** neuronal ceroid lipofuscinosis, *CLN2*, optical coherence tomography, automated segmentation, parafovea, retinal degeneration

## Abstract

**Purpose:**

*CLN2-*associated disease is a hereditary, fatal lysosomal storage disorder characterized by progressive brain and retinal deterioration. Here, we characterize the inner and outer retinal degeneration using automated segmentation software in optical coherence tomography scans, providing an objective, quantifiable metric for monitoring subtle changes previously identified with a validated disease classification scale (the Weill Cornell Batten Scale).

**Methods:**

This study is a retrospective, single-center cohort review of images from examinations under anesthesia in treatment-naïve patients with *CLN2*-associated disease. Automated segmentation software was used to delineate retinal nerve fiber, ganglion cell layer (GCL), and outer nuclear layer (ONL) thickness measurements in the fovea, parafovea, and perifovea based on age groups (months): 30 to 38, 39 to 45, 46 to 52, 53 to 59, 60 to 66, and 67 or older.

**Results:**

Twenty-seven eyes from 14 patients were included, with 8 serial images yielding 36 interpretable optical coherence tomography scans. There was a significant difference in parafoveal ONL thickness between 39 to 45 and 46 to 52 months of age (*P* = 0.032) not seen in other regions or retinal layers. Perifoveal ONL demonstrated a difference in thickness between the 60 to 66 and greater than 67 months age cohorts (*P* = 0.047). There was strong symmetry between eyes, and high segmentation repeatability.

**Conclusions:**

Parafoveal ONL thickness represents a sensitive, early age indicator of *CLN2*-associated degeneration. Outer retinal degeneration is apparent at younger ages than inner retinal changes though in treatment-naïve patients all retinal layers showed significant differences between 60 to 66 and more than 67 months of age.

**Translational Relevance:**

This study establishes sensitive, quantitative biomarkers for assessing retinal degeneration in a large cohort natural history study in anticipation of future clinical trials.

## Introduction

Neuronal ceroid lipofuscinosis (NCL) is a collection of distinct lysosomal storage diseases that, although rare, represents the most common progressive neurodegenerative childhood disease.^1^^–^^4^ Currently, classification of NCL is based on specific genes (*CLN,* ceroid lipofuscinosis, neuronal) and mutations involved, and the age at clinical manifestation.^5^ Each *CLN* gene is distinctly numbered to designate a respective subtype, of which there are currently 14 known genes affected.^6^ The most common subtype is associated with mutations in *CLN2*, also previously called Jansky–Bielschowsky disease or CLN2 disease.^7^ *CLN2* encodes for tripeptidyl peptidase 1 (TPP1), a lysosomal hydrolase that removes three amino acids from the *N*-terminus of small polypeptides.^8^^,^^9^ As a result, mutations in *CLN2* lead to the accumulation of curvilinear profiles in lysosomal residual bodies throughout the body, to which the central nervous system (CNS) and retina seem to be particular sensitive.^10^ Patients often present between 2 and 4 years of age with delayed acquisition or deterioration of speech, motor decline, seizures, and ataxia, with a more pronounced and rapidly progressive deterioration of motor function, language, vision, and swallowing after age 3. The disease typically leads to premature death by early adolescence, although naturally the timing and course depends on the specific mutation.^11^^–^^14^

CLN2 is also the only NCL currently with a pharmacologic intervention approved in the United States and European Union. Treatment consists of enzyme augmentation therapy with cerliponase alfa, a recombinant human proenzyme of TPP1, administered every second week by intracerebroventricular infusions via a reservoir surgically implanted under the scalp. This therapy works in slowing the progressive motor and speech decline by decreasing the accumulation of toxic lysosomal storage material.^15^,^16^ These infusions do not treat the primary outer retinal degeneration, which continues unabated.^17^^,^^18^

In *CLN2*-associated disease, vision loss tends to occur late in the disease course after other symptoms have become manifest; however, retinal structural changes can be noted on optical coherence tomography (OCT) in patients as young as 2 years of age.^19^ As such, OCT has been recommended in *CLN2*-associated disease management guidelines to monitor retinal changes owing to its sensitivity (and that invasive with fluorescein and indocyanine green angiography are unnecessary).^20^ Prior studies have reported on multimodal imaging characterizing retinal findings in patients’ with confirmed *CLN2* mutations, established and validated a scoring system (Weill Cornell Batten Severity Score [WCBS]),^21^ as well as a delineated the natural history and symmetry of the disease in anticipation of future retina-specific therapeutic interventions.^22^ However, most measurements of the degeneration as delineated by OCT (including in our own prior work) focused on central macular thickness measurements, which may not be the most accurate means of accurately classifying the degeneration. Classically, the retinopathy associated with CLN2 disease manifests as a “bull's eye” maculopathy and atrophy of the retinal pigment epithelium, autofluorescence changes, peripheral retinal pigmentary changes, and marked reduction in electroretinogram.^23^^–^^27^ Critically, the disease process is first identifiable in the parafovea (defined as a 2.5-mm diameter ring centered on the fovea), progress to involve the fovea (1.5-mm diameter ring), and then last involve the perifovea (5.5-mm diameter ring centered on the fovea). The outer retinal changes begin with subtle attenuation followed sequentially by frank loss of the ellipsoid zone on OCT scans. We hypothesized that with segmentation of the retinal layers, these subtle parafoveal changes would be quantitatively discernable before most current conventional means of tracking *CLN2-*associated retinal degeneration.

The concept of assessing parafoveal degeneration with segmentation software in general for bull's eye maculopathies is not novel. Prior studies have sought to use either commercially available or customized software to assess parafoveal loss of outer versus inner retinal layers for hydroxychloroquine retinal toxicity, which has a well-established and discernable predilection for parafoveal OCT changes.^28^^–^^31^ Other work has suggested that for hydroxychloroquine toxicity the outer nuclear layer (ONL) thinning may precede parafoveal ellipsoid disruption, or continue after cessation.^32^^,^^33^ As such, perhaps subtle thinning of the ONL as measured by automated segmentation in CLN2 disease may precede more advanced photoreceptor outer segment degeneration and thus serve as a sensitive biomarker for disease onset, progression, and response to treatment. In particular, the ability to monitor the area of the retina precisely, and the concordant time course of degeneration in each area, is critical in the setting of possible future intervention, either enzyme replacement or gene therapy.

In the present study, we sought to use commercially available automated segmentation software (Heidelberg Engineering, Heidelberg, Germany) to comprehensively delineate the outer versus inner retinal degeneration associated with CLN2 disease in patients not concurrently receiving CNS therapy. As such, the goals of the study were to (1) determine the feasibility and repeatability of commercially available segmentation software in quantitatively measuring retinal layers by anatomic zone in *CLN2*-associated retinal degeneration; (2) objectively capture the age at which significant parafoveal manifestations of the degeneration may occur; and (3) describe any age-associations of the degeneration in the inner and outer retinal layers. Such precise quantitative modeling should help to provide metrics for future retina-specific early phase clinical trials in these patients.

## Methods

Fourteen treatment-naïve patients with CLN2 disease, genetically confirmed to have mutations in *CLN2*, underwent a comprehensive ophthalmic examination under anesthesia as a part of their participation in a natural history trial at Weill Cornell Medical College and New York/Presbyterian Hospital (NCT01035424). The study protocol was approved by the Institutional Review Boards of Weill Cornell Medical College and New York/Presbyterian Hospital. The study was conducted in accordance with the principles of the Declaration of Helsinki and the International Conference on Harmonisation Good Clinical Practice guidelines. Informed consent was obtained for all patients before enrollment. These patients are a subset of the same cohort previously reported as part of a retinal natural history report^22^; however, only those with adequate imaging were included in the present study (imaging specifications are provided elsewhere in this article).

### Examinations and Patient Information

Patients were referred to our center after undergoing genetic testing to confirm that patients were bi-allelic with at least one of the following mutations: C3670T (c.622 C>T, nonsense Arg208 to stop), G3556C (c.509-1G>C, intron 5, splice), G4655A (c.1094G>A, Cys365Tyr), or G1950C (c. 229 G>C, Gly77Arg). Testing was performed in multiple laboratories depending on the origin of the referral. As a result, not all laboratories were likely Clinical Laboratory Improvement Amendments certified; however, our institution did not specifically verify this status. All patients referred to our center were included in the natural history trial (ie, no patients were excluded); however, only those patients who had OCT images of sufficient quality to accurately measure segmented retinal layer thicknesses were included in this analysis. Two pairs of patients included in the final analysis were siblings.

The ocular examinations under anesthesia were conducted as part of a natural history study which supported clinical trial sponsored by the National Institutes of Health before the initiation of any study-related treatment. All patients were completely treatment naïve at each time point in the study.

Ocular examinations were performed during general anesthesia, which was necessary for patients with severe motor and cognitive abnormalities. Comprehensive assessments were performed as previously reported.^21^^,^^22^ For the present study, all included patients had imaging with spectral domain OCT (Heidelberg Engineering). Gathered clinical information included age at time of diagnosis, age at time of examination, gender, and laterality of eye being examined. Twenty-seven eyes from 14 patients had adequate OCT images obtained.

### Image Acquisition and Segmentation

During the examinations under anesthesia, patients were positioned supine and the Heidelberg Spectralis camera detached from the chinrest and held orthogonal to the pupillary plane for image acquisition. OCT images were acquired with the Heidelberg Spectralis fast macular protocol consisting of 25 B scans with 512 A scans. Only those OCT images with sufficient signal quality for segmentation analysis centrally, parafoveally, and perifoveally were included in the analysis, as deemed by gross abnormalities in segmentation references lines when reviewed by a single image grader (KDK). As such, some OCT images reported in prior reports on this cohort were not included owing to inadequate segmentation capabilities of the aforementioned regions.

**Figure 1.**
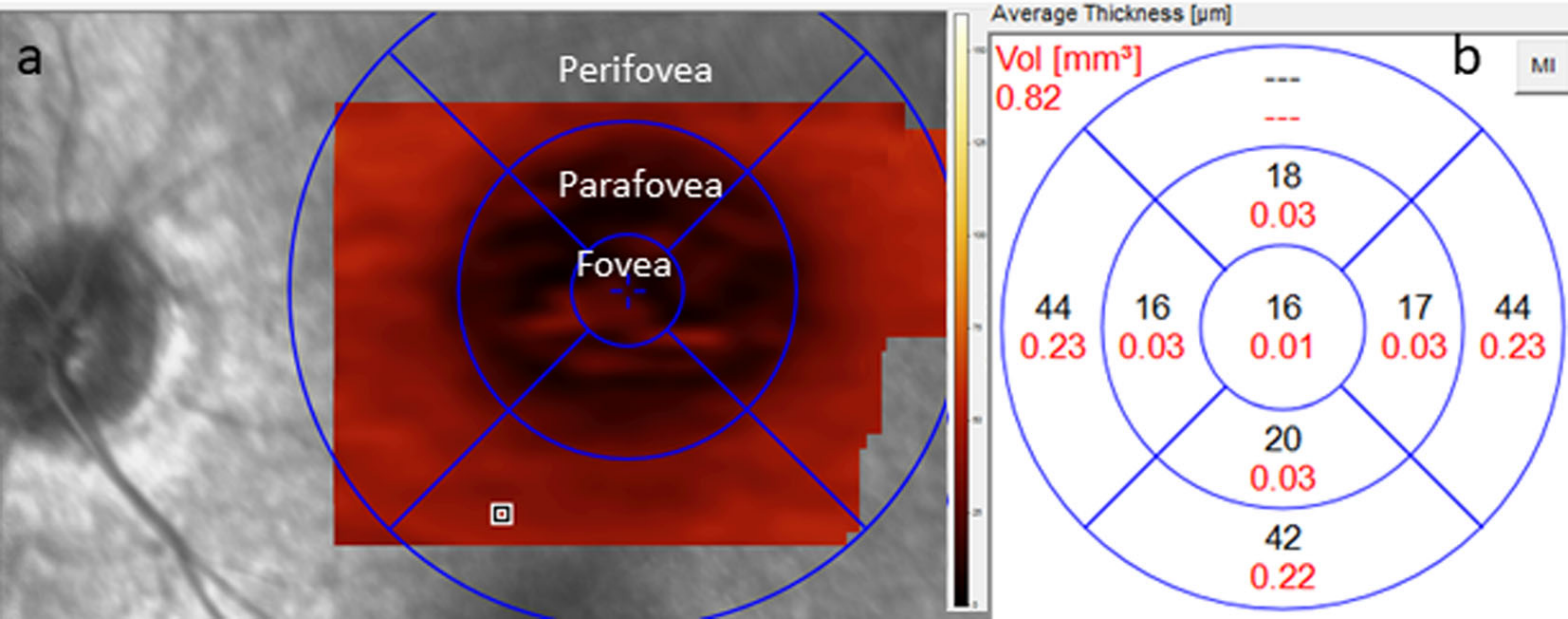
Representative diagram of automated segmentation output and measurements, with this specific example demonstrating en face outer nuclear layer (ONL) thickness. Standardized ETDRS reference circles of 1, 3, and 6 millimeter diameter were centered on the fovea (a), approximating the foveal, parafoveal, and perifoveal regions. Adjacent color scale of thickness in micrometers (µm) for the thickness map. For each region a thickness average is generated by the software (b). For ONL measurements, three regional measurements were compiled: (1) foveal, from the 1-mm ETDRS circle, (2) parafoveal, an average of the four measurements from the 3-mm ETDRS circle, and (3) perifoveal, an average score of the four measurements from the 6-mm ETDRS circle. This example also demonstrates how one perifoveal quadrant (superior) lacked sufficient thickness measurements to generate an average output.

For each eye on each examination date, images had automated segmentation lines generated by the Heidelberg Spectralis software for each B scan raster of a macular cube scan. Scans with obvious alignment errors across all retinal layers were excluded from analysis. Scans in which a single segmentation line was found to be out of position owing to artifact within the OCT image were manually re-placed by a single image grader (KDK, masked to all clinical information including age and WCBS scores) to align with presumptive correct anatomy (see Supplementary Fig. S1 demonstrating artifact and adjusted measurements). Given that our patients were anesthetized and unable to cooperate, the images obtained were unable to prospectively account for previously described artifact from Henle's fiber layer based on OCT entry beam angle through the pupil.^34^ Manual adjustments were made by a single masked grader (KDK) during image processing in seven images: three to account for this observable hyper-reflectivity in Henle's layer and four for the loss of identifiable retinal layers subfoveally in eyes with advanced degenerative findings. Images were then assessed via retinal thickness mapping of the various segments with 1-, 3-, and 6-mm standardized Early Treatment Diabetic Retinopathy Study (ETDRS) diameter rings as preset by the Heidelberg Spectralis software (Fig. 1), approximating the regions of the fovea, parafovea, and perifovea. This grid was aligned manually with the foveal center when scans were found to be taken off-center. Thickness measurements were recorded for nerve fiber layer (NFL), ganglion cell layer (GCL), and ONL (outer plexiform layer to external limiting membrane) in each regional domain, including the fovea, four parafoveal regions, and four perifoveal regions. For analysis, a single composite parafoveal and perifoveal thickness value was generated for each layer by averaging the four values from each respective region (ie, both a composite parafovea and perifovea value). In scans where one region of the ETDRS grid had insufficient data to generate a thickness average (usually perifoveal), the composite thickness for the region was comprised of an average of the other three regions. There were no scans with more than one region without a thickness measurement. Scans from each time point for each eye were all included in the longitudinal analysis. The central subfield thickness (CST) was also recorded within the 1-mm ETDRS diameter ring for each scan.

Test–retest reliability was assessed in eyes that had more than one usable fast macular volume scans. The same segmentation and measurement protocols were repeated in a blinded fashion (the grader was unaware of prior scan values, patient age, and WCBS score).

### Statistical Analysis

OCTs were binned into various cohorts bracketed by age based on anticipated time points of degeneration onset from prior studies: 30 to 38 months, 39 to 45 months, 46 to 52 months, 53 to 59 months, 60 to 66 months, and 67 months and older.^21^^,^^22^ These represent 6-month intervals starting just before (hypothesizing that segmentation measurements may prove more sensitive than previously used conventional degeneration parameters like retinal thickness) and running through a previously identified critical period in the retinal degeneration between 48 and 72 months of age. Measurements for the various retinal layer thickness measurements were compared between adjacent age cohorts using a mixed effect model analysis of variance, treating right and left eyes from the same patient as repeat observations. Tukey's multiple comparison test was used given the use of multiple stepwise comparisons. Pearson correlation coefficients were used to assess thickness measurements with age at time of examination (nonbinned), and to assess right and left eye measurement correlations. For all correlations, a *P* value of less than .05 was deemed to be significant. Intraclass correlation coefficients (ICC) were used to assess the test–retest reliability of repeated scans. Analysis of variance correlations were performed with GraphPad Prism version 9.0 for Windows (GraphPad Software, San Diego, CA) and Pearson correlations and intraclass correlation coefficients were performed with SPSS statistics for Windows version 25.0 (IBM Corp., Armonk, NY).

## Results

Patients had a mean age of 42 *±* 13.7 months at the time of diagnosis and 57.2 *±* 18.0 months at the time of first examination (Table 1). Ten patients (71.4%) were female. For the purposes of correlating age cohorts with disease state the following OCT scans were available: 27 eyes from 14 treatment-naïve patients underwent at least one examination with interpretable macular-cube OCT scans; six eyes from three patients underwent two examinations; and two eyes from one patient underwent three examinations (Table 2). For those patients with serial examinations there was a mean duration of follow-up (from first to last examination) of 4 *±* 3.1 months. There were 36 interpretable OCT scans for the entire cohort out of 44 total OCT scans. It was possible to generate ICCs based on multiple interpretable scans in 12 of 36 eyes. Across all regions (fovea, parafovea, and perifovea) ICCs were 0.971 for total retinal thicknesses, 0.923 for NFL thicknesses, 0.959 for GCL thicknesses, and 0.951 for ONL thicknesses.

**Table 1.** Baseline Demographics, Imaging, and Follow-up of Patient Cohort (*N* = 14)

Characteristics	Number or Mean ± SD	Percent or Range
Female sex	10	71.4%
Mean age at diagnosis (months)	42 ± 13.7	18–56
Mean age at first examination (months)	57.2 ± 18.0	30–97
Mean serial examination follow-up (months)	4 ± 3.1	1–8
Subjects with serial OCT	4	28.6
Total number of OCT scans included	36	–

SD, standard deviation.

**Table 2.** Serial OCT Scans and Patient Age

	First	Second	Third
Patient	OCT Age	OCT Age	OCT Age
1	81	–	–
2	59	–	–
3	65	–	–
4	63	–	–
5	39	41	–
6	52	53	60
7	53	–	–
8	32	33	–
9	55	–	–
10	65	70	–
11	47	–	–
12	63	–	–
13	97	–	–
14	30	–	–

All ages in months.

Representing a wide breath of disease states based on age, OCT scans were distributed across the time intervals (Table 3), with five scans between 30 and 38 months, four scans between 39 and 45 months, four scans between 46 and 52 months, seven scans between 53 and 59 months, ten scans between 60 and 66 months, and six scans at more than 67 months of age.

**Table 3.** Number of OCT Scans Obtained per Time Period

Age at Time of Examination (Months)	OCT Scans
30–38	5
39–45	4
46–52	4
53–59	7
60–66	10
≥67	6

All segmented OCTs were inversely correlated with patient age (Table 4), confirming a strong inverse age association occurring in both the inner and outer retina, as previously described.^22^ Pearson correlations between right and left eye segmentation measurements were positively correlated, demonstrating disease symmetry, also as previously described,^22^ with one scan excluded in analysis because it had no partner image (Table 4). In particular, the parafoveal and perifoveal ONL measurements had remarkable right–left correlation (Pearson coefficient 0.99 for both). The only nonsignificant correlation in symmetry was in the parafoveal NFL (0.48; *P* = 0.089).

**Table 4.** Age at Time of Examination and Laterality Pearson Correlations

	Age–Thickness		Right–Left	
	Correlation	*n* = 36	Thickness Correlation	*n* = 17
CST	–0.72	<0.001	0.56	0.017
Parafoveal NFL	–0.63	<0.001	0.48	0.089
Parafoveal GCL	–0.55	<0.001	0.95	<0.001
Foveal ONL	–0.79	<0.001	0.51	0.031
Parafoveal ONL	–0.75	<0.001	0.99	<0.001
Perifoveal ONL	–0.74	<0.001	0.99	<0.001

GCL, ganglion cell layer; CST, central subfield thickness; NFL, nerve fiber layer; ONL, outer nuclear layer.

**Figure 2.**
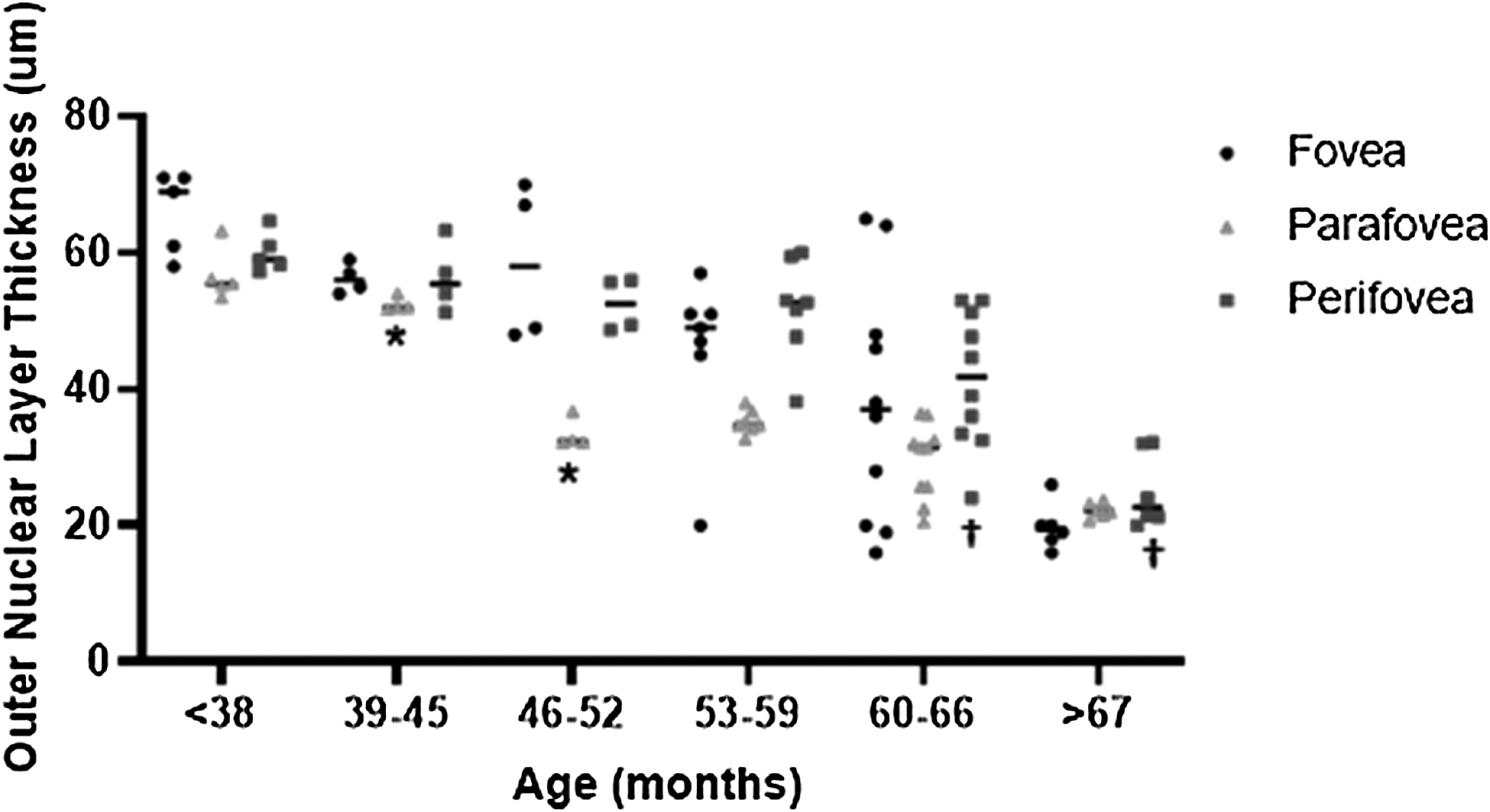
Graph showing each scan measurement and mean for fovea (*circle*), parafovea (*triangle*), and perifovea (*square*) outer nuclear layer (ONL) thickness by age at time of examination cohorts (months). Horizontal dashes represent the mean for each age cohort measurement. Adjacent age cohorts from the same region were compared using mixed effect analysis of variance with Tukey's multiple comparison test. There was a significant difference (*P* = 0.032) in parafoveal thickness between 39 to 45 months and 46 to 52 months of age (*) that did not have a correlating significant difference in foveal or perifoveal ONL thickness. Only the perifovea demonstrated a significant difference in thickness between 60 to 66 and more than 67 months of age (**†***P* = 0.047).

**Figure 3.**
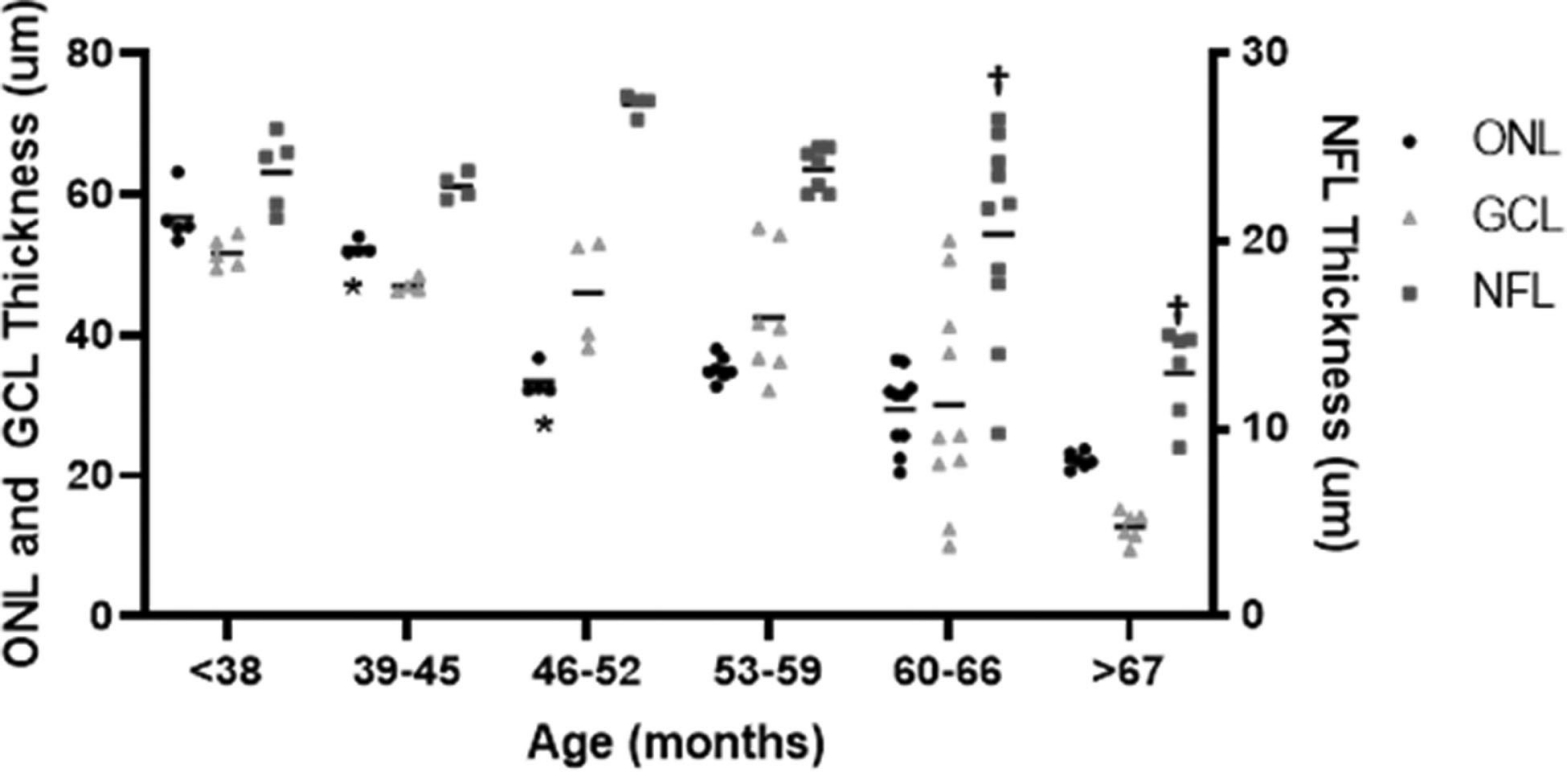
Graph showing each scan measurement of parafoveal thickness measurements of the outer nuclear layer (ONL) (*circle*), ganglion cell layer (GCL) (*triangle*) and nerve fiber layer (NFL) (*square*) by age at time of examination cohorts (months). Horizontal dashes represent the mean for each age cohort measurement. Adjacent age cohorts from the same retinal layer were compared using mixed effect analysis of variance with Tukey's multiple comparison test. There was a significant difference (*P* = 0.032) in parafoveal ONL thickness between 39 to 45 months and 46 to 52 months of age (*). Inner retinal thinning (GCL and NFL) did not demonstrate statistically significant differences between cohorts, except for a borderline trend for the NFL between 60 to 66 months and more than 67 months of age (**†***P* = 0.053). NFL measurements are plotted on secondary axis (*right side*).

There was a significant difference in parafoveal ONL thickness between the 39 months and 46 months time intervals (*P* = 0.032), which was not reflected in the foveal and perifoveal ONL measurements (Fig. 2). There was a significant difference in thickness measurements for only the perifoveal ONL region between the 60 months and greater than 67 months time intervals (*P* = 0.047). Although there were differences between measurements in the parafoveal inner retina (NFL and GCL) between sequential age cohorts (Fig. 3), none of these differences were significant until a borderline difference in the NFL between the 60- to 66-months and 60- to 67-months cohorts (*P* = 0.053).

Unlike segmented thickness measurements, the CST did not demonstrate a statistically significant difference in thickness between any age cohorts, but did show some trend toward significant difference in CST between measurements from the 53 months and 60 months age cohorts (*P* = 0.089) and between the 60 months and greater than 67 months cohorts (*P* = 0.137).

## Discussion

The current analysis focused on using retinal layer segmentation software to assess *CLN2*-associated retinal disease. In the context of this rare disease, this study with 36 scans from 14 patients represents a sizeable cohort of patients to characterize the distinct anatomic features of the degeneration and identify certain biomarkers that may prove more sensitive than those used in prior studies (such as the CST). In this cohort of untreated patients, our findings suggest a significant parafoveal ONL degeneration that is apparent in younger patients, which occurs at younger ages than significant differences in the fovea or perifoveal ONL, with significant differences in inner retinal degeneration occurring at more advanced ages. Importantly, parafoveal ONL thinning seems to be one of the earliest objective findings in *CLN2*-associated retinal degeneration, occurring before the critical period identified by more conventional measurements like CST (identified as 48–72 months in prior work),^22^ and also preceding frank ellipsoid zone loss. There is also remarkable disease symmetry between eyes across the various segmented layers of the retina, as has previously been reported, with Pearson correlation coefficients ranging from 0.48 to 0.99. It is worth mentioning that such disease symmetry is not present in other genetic related retinal degenerations such as Stargardt's disease (17.6% discordant progression rates) or retinitis pigmentosa (up to 20% with significant disease asymmetry).^35^^,^^36^ Thus, this study simultaneously verifies work from previous studies regarding the disease pathology^22^ and also confirms the repeatability of the segmentation process, particularly for the highly concordant ONL measurements.

**Figure 4.**
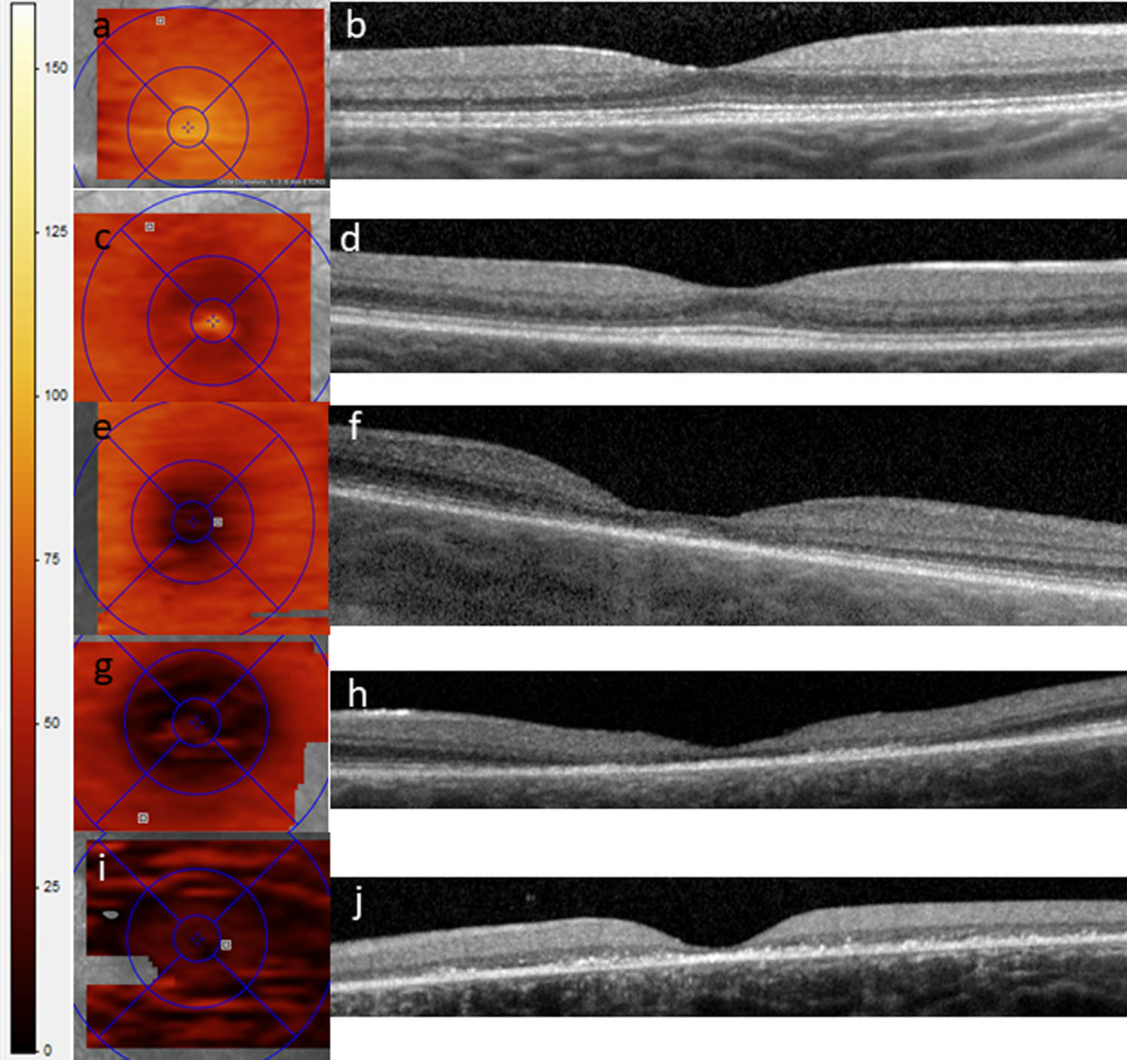
En face OCT scans of the outer nuclear layer (ONL) with ETDRS 1-, 3-, and 6-mm overlay (a, c, e, g, i) compared with transfoveal OCT B scans from the same eye (b, d, f, h, i). Each pair of images represents successively more advanced degeneration as categorized by Weill Cornell Batten scale. WCBS 1 (a, b) represents essentially no anatomic findings of degeneration. WCBS 2 (c, d) shows subtle early parafoveal outer nuclear thinning without ellipsoid or external limiting membrane loss. WCBS 3 (e, f) demonstrates robust parafoveal outer nuclear thinning with loss of parafoveal ellipsoid, but still preservation of some ellipsoid subfoveally. WCBS 4 (g, h) demonstrates parafoveal and foveal ONL loss with almost complete outer retinal loss subfoveally WCBS 5 (i, j) shows more global ONL loss expanding outwards from the fovea, now including the perifovea. Left side of image shows color scale of thickness in micrometers (µm) for the thickness maps in (a, c, e, g, i).

It is worth noting that the observed sequence of ONL changes directly correlates with the various stages of the degeneration as delineated by the WCBS,^21^^,^^22^ and is readily visible in the en face ONL OCT images (Fig. 4). Specifically, measurement of the ONL automatically and objectively characterizes the transition from score 1 (no findings) to score 2 (mild early findings), which would otherwise require a subjective interpreter, rather than an automated, commercially available segmentation tool, to evaluate for subtle attenuation of the parafoveal ellipsoid zone on OCT. These changes occur before true loss of the ellipsoid zone, which increasingly reflects later stages of the outer retinal degeneration. Gene therapy has demonstrated slowing of the neurological progression of CNS CLN2 disease by CNS administration of an adeno-associated serotype rhino vector mediating expression of the *CLN2* gene^37^; however, there will likely be need for a retina-specific intervention as well, given the independent outer retinal degeneration in these patients.^38^,^39^ As such, ONL segmentation provides a noninvasive and more objective metric of disease severity than the subjective grading scale, and likely a more accurate quantitative measure of disease state in the event of this future intervention directed at the retinal degeneration. Importantly, measurements were reliably repeatable, with high ICC for all segmentation layers when multiple volume OCT scans were obtained at the time of the initial examination.

There have been previous reports on OCT imaging using this same cohort of patients,^22^ but all modes of measurement except for CST were conducted differently in the present study. The prior study evaluated NFL thickness at a single point 3 mm nasal to the fovea center as measured manually by a single observer, rather than diffusely as part of automated segmentation software, and found no correlation of thickness with age. The present study represents more reliable measurements of the NFL, and thus that the inverse age association of NFL measurements with increasing age is likely representative of disease characteristics in treatment naïve patients. In contrast with that of the ONL, NFL measurements were found to be less concordant between eyes, which we hypothesize to be due to the “thin” measurements meaning single micrometer discrepancies would yield high variability.

None of the patients in this current cohort had received any form of CNS-directed therapy. As such, there is some expected differences in inner retinal layer thicknesses, in particular the difference in NFL thicknesses between the more advanced age cohorts. It is unclear if this finding reflects secondary degeneration from the primary outer retinal degeneration or independent from the retinal process and related to the CNS degeneration. There are other studies that affirm that the retinal degeneration continues independent from CNS degeneration, but that does not necessarily indicate causality for changes in the inner retina.^15^^,^^40^ Although this study is not powered to specifically answer that question, the findings do suggest that significant inner retinal loss occurs at a more advanced age (after 60 months age) than the significant changes in outer retinal layers. Further studies are certainly necessary, and of specific interest would be segmented analysis of OCTs from a cohort of patients treated with enzyme augmentation therapy, ceronolipase-alpha,^15^ or gene therapy,^33^ to the CNS.

All of the enrolled patients were part of the screening study for a phase I CNS gene therapy trial; therefore, there could be enrollment bias based on the severity of the disease as families sought experimental treatment options. However, this study includes patients ranging from 30 to more than 97 months of age, similar to previous reports on this cohort, demonstrating imaging findings across a wide spectrum of disease states.

As a retrospective study with a fixed cohort and set of images to draw upon, there are a number of other limitations for the present study. First, scans were used from both eyes for each patient. This strategy was necessary owing to the small number of total scans available; however, given the disease symmetry in these patients, as prior work has established and this study further confirms,^22^ it does potentially over-represent specific *CLN2*-associated degeneration phenotypes present in this cohort. This analysis also assumes consistency in the phenotypic manifestations of a variety of mutations in *CLN2*; however, our prior studies on this cohort of patients found no difference between genotypes,^21^ and also strong consistency in the stage of degeneration at a particular age.^22^ Also, only four patients (eight eyes) had serial examinations, and as such the natural history of the changes on OCT assumes similar disease progression rates with advancing age across patients and different mutations. This assumption may not be entirely valid, but other work with serial visits assessing nonvisual disease progression did find similar disease progression aligned with those in the current study.^41^ Although a large cohort of patients with *CLN2-*associated degeneration, the samples are still statistically small and within any population there is a normative distribution of retinal thicknesses. Further work is needed to contribute more longitudinal analysis or possibly with larger cohorts to validate the differences in thickness measurements. All patients observed in this study were participants in a natural history study whose primary end point was not observations of the retinal degeneration. Regular longitudinal assessments would be ideal for following the retinal disease, but these examinations require anesthesia owing to the limited ability of these patients to productively participate in outpatient clinical examinations. Likewise, this study is purely anatomic and does not assess visual function in these patients. As with all imaging studies of novel biomarkers, there is always the challenge of validating novel measurements.

OCT scans in this study were obtained by manually holding the Heidelberg Spectralis device in appropriate position over a supine patient, but future studies may benefit from the use of the FLEX module from Heidelberg (which was released after the duration of the present study) to better standardize image acquisition. Some OCT scans (7 of the 36 total) required manual adjustment of the automated segmentation lines, which introduces the possibility of bias and variability into the final outcome. Four of these corrections were subfoveal in the setting of advanced disease, and three were in the setting of increased parafoveal hyper-reflectivity in Henle's layer in patients with early/minimal disease. It is worth noting that the perifovea regional measurements were being made, on average, with slightly less data compared with that from the fovea and parafovea. The perifovea was more commonly missing values in one quadrant compared with the parafovea; however, there did not seem to be a major difference in the variability between regional measurements in the perifovea versus parafovea (as seen in the data distribution in Fig. 2). Future studies would seek to improve the automated segmentation software in the setting of artifact or advanced pathology, or to demonstrate interobserver repeatability with scan adjustments. Nonetheless, evaluation of retinal disease degeneration via an automated segmentation algorithm using standardized OCT measurements likely introduces more consistency and precision, as indicated by the high intraclass correlation coefficients (ICCs) in our study, and less bias compared with observer bucketing of OCT scans into the previously described WCBS.

In summary, the present study sought to further characterize the anatomic features of *CLN2*-associated retinal degeneration using commercially available automated segmentation software. We found significant changes in the parafoveal ONL between young age cohorts that represent an objective measure of a previously validated qualitative assessment (WCBS), which may serve as a more sensitive biomarker for the onset of degeneration compared to other OCT measurements such as CST. The automated segmentation allowed for demonstration of early outer retinal degeneration, specifically in the parafovea, with significant differences in the inner retina occurring between more advanced age cohorts. Our findings demonstrate the potential capacity of OCT to provide objective biomarkers in monitoring *CLN2-*associated retinal degeneration, critical in the context of potential future retina-specific interventions like gene therapy. Further research in larger cohorts would be ideal, but is limited by the relatively small patient population, and therapeutic interventions can be followed with the study of the inner versus outer retinal degeneration rates in patients undergoing concurrent CNS therapy.

## Supplementary Material

Supplement 1
