# Supplementary material for: Automated Retinal Layer Segmentation in CLN2-Associated Disease: Commercially Available Software Characterizing a Progressive Maculopathy
Source: Transl Vis Sci Technol. 2021 Jul 27;10(8):23. doi: 10.1167/tvst.10.8.23 (PMC8322716; doi:10.1167/tvst.10.8.23)
Supplement: Supplement 1 [file tvst-10-8-23_s001.pdf]

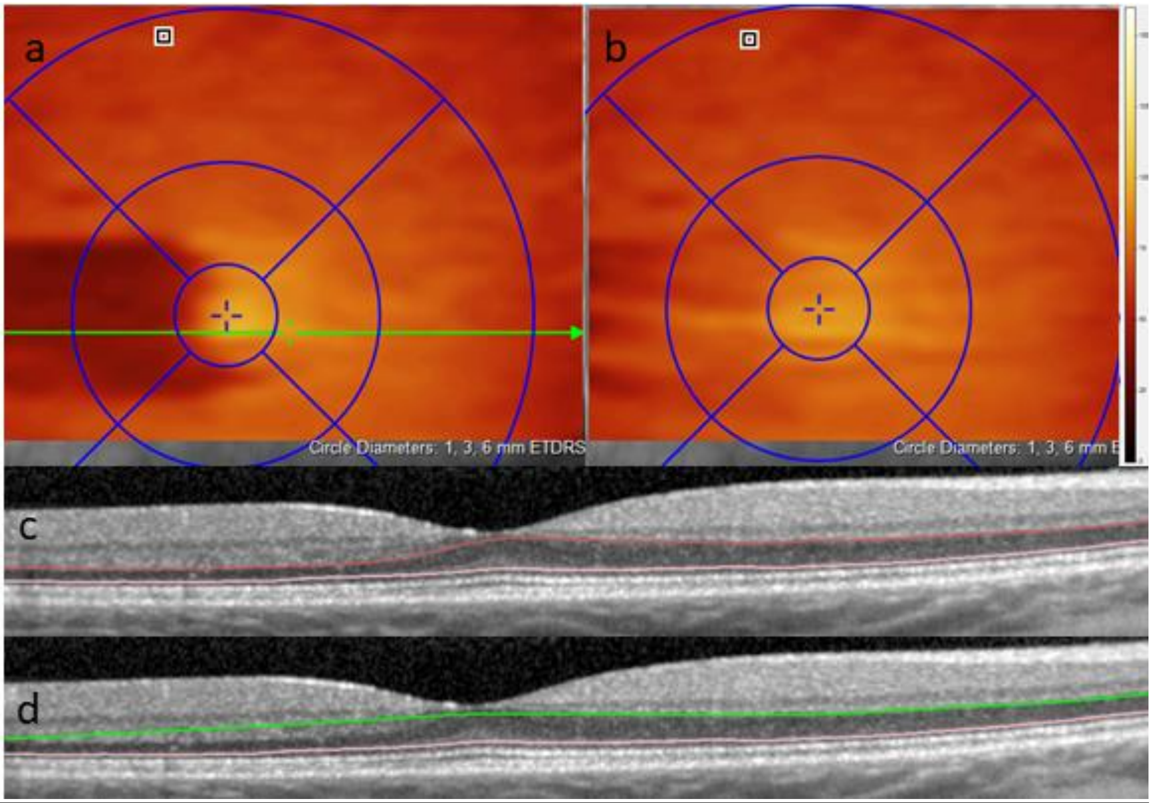

Supplemental Figure 1:

Enface OCT image of outer nuclear layer (ONL) before (a) and after (b) manual correction of segmentation accounting for temporal parafoveal hyper-reflectivity of Henle's layer. OCT B scans (c) demonstrating automated segmentation of the ONL between outer plexiform layer (upper boundary) and external limiting membrane. Due to temporal parafoveal hyperreflectivity in Henle's layer, manual correction of the upper boundary was performed in the Heidelberg software yielding corrected segmentation (d). Adjacent color scale of thickness in micrometers (μm) for the thickness maps in (a) and (b). This patient was aged 30 months at the time of the examination and was WCBS score 1.
